# MRI characteristics predict risk of pathological upgrade in patients with ISUP grade group 1 prostate cancer

**DOI:** 10.1007/s00330-024-11062-2

**Published:** 2024-09-13

**Authors:** M. Boschheidgen, L. Schimmöller, J. P. Radtke, R. Kastl, K. Jannusch, J. Lakes, L. R. Drewes, K. L. Radke, I. Esposito, P. Albers, G. Antoch, T. Ullrich, R. Al-Monajjed

**Affiliations:** 1 Department of Diagnostic and Interventional Radiology, University Dusseldorf, Medical Faculty, D-40225 Dusseldorf, Germany; 2 Department of Diagnostic, Interventional Radiology and Nuclear Medicine, Marien Hospital Herne, University Hospital of the Ruhr-University Bochum, Herne, Germany; 3 Department of Urology, University Dusseldorf, Medical Faculty, D-40225 Dusseldorf, Germany; 4 Department of Pathology, University Dusseldorf, Medical Faculty, D-40225 Dusseldorf, Germany

**Keywords:** Prostatic neoplasms, Multiparametric magnetic resonance imaging, Risk stratification, Low-risk prostate cancer

## Abstract

**Objective:**

This study aims to analyse multiparametric MRI (mpMRI) characteristics of patients diagnosed with ISUP grade group (GG) 1 prostate cancer (PC) on initial target plus systematic MRI/TRUS fusion-guided biopsy and investigate histopathological progression during follow-up.

**Methods:**

A retrospective single-centre cohort analysis was conducted on consecutive patients with mpMRI visible lesions (PI-RADS ≥ 3) and detection of ISUP-1-PC at the time of initial biopsy. The study assessed clinical, mpMRI, and histopathological parameters. Subcohorts were analysed with (1) patients who had confirmed ISUP-1-PC and (2) patients who experienced histopathological upgrading to ISUP ≥ 2 PC during follow-up either at re-biopsy or radical prostatectomy (RP).

**Results:**

A total of 156 patients (median age 65 years) between March 2014 and August 2021 were included. Histopathological upgrading to ISUP ≥ 2 was detected in 55% of patients during a median follow-up of 9.5 months (IQR 2.2–16.4). When comparing subgroups with an ISUP upgrade and sustained ISUP 1 PC, they differed significantly in contact length of the index lesion to the pseudocapsule, ADC value, PI-RADS category, and the MRI grading group (mGG) (*p* < 0.05). In the ISUP GG ≥ 2 subgroup, 91% of men had PI-RADS category 4 or 5 and 82% exhibited the highest mGG (mGG3). In multivariate analysis, mGG was the only independent parameter for predicting ISUP ≥ 2-PC in these patients.

**Conclusions:**

MRI reveals important information about PC aggressiveness and should be incorporated into clinical decision-making when ISUP-1-PC is diagnosed. In cases of specific MRI characteristics adverse to the histopathology, early re-biopsy might be considered.

**Clinical relevance statement:**

In cases with clear MRI characteristics for clinically significant prostate cancer (e.g., mGG 3 and/or PI-RADS 5, cT3, or clear focal PI-RADS 4 lesions on MRI) and ISUP GG 1 PC diagnosed on initial prostate biopsy, MRI findings should be incorporated into clinical decision-making and early re-biopsy (e.g., within 6 months) might be considered.

**Key Points:**

- *MRI reveals important information about prostate cancer (PC) aggressiveness*.
- *MRI should be incorporated into clinical decision-making when ISUP GG 1 PC is diagnosed on initial prostate biopsy*.
- *In cases of specific MRI characteristics adverse to the histopathology, early re-biopsy might be considered*.

**Graphical Abstract:**

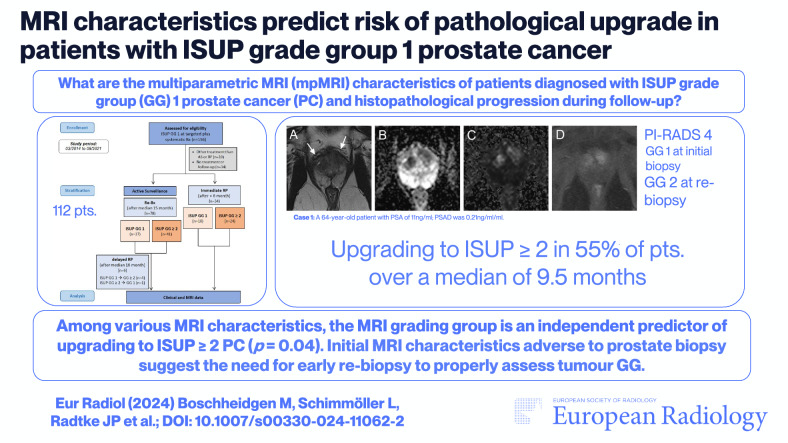

## Introduction

Multiparametric MRI (mpMRI) of the prostate has become a fundamental part of the diagnostic pathway for patients with suspicion of prostate cancer (PC). Its sensitivity in detecting clinically significant cancer reaches up to 95% [1]. Furthermore, it reveals important information on PC aggressiveness and can reliably identify patients with higher-risk PC, who require active treatment [2–4]. On the other hand, lower-grade PC is frequently non-visible on mpMRI. In those scenarios, active surveillance (AS) is the recommended treatment of choice due to the indolent nature of these tumours [5]. Even though already the initial mpMRI may differentiate between low-risk and high-risk disease, it is so far not recommended as a selection criterion in AS protocols due to high variability in readers’ experiences and biopsy techniques. This may be improved by correct labelling of the index lesion in imaging data, by optimising the biopsy system, by standardised training of biopsy procedures, and finally, by reference pathology [6]. Previous publications have indicated that limited MRI quality elevates diagnostic uncertainty and contributes to the understaging of PC (PC) in MRI evaluations, so image quality is also crucial for excellent diagnostic value [7, 8].

Numerous studies have shown that among patients eligible for AS, upstaging and upgrading in postoperative pathology reports account for up to 47% and 60% of cases, respectively [9, 10]. Nevertheless, this data is mainly based on systematic biopsy [10]. Large multicentre studies for the evaluation of MRI-guided biopsies in AS cohorts are still pending [2]. The European Association of Urology guidelines recommend performing mpMRI before the confirmatory biopsy in AS management, with biopsy being repeated at least every 2–3 years [5, 11]. This causes patient compliance problems due to inconvenience [12]. However, there is evidence that mpMRI and MRI/TRUS-fusion biopsy prior to inclusion in AS increase histopathological accuracy and consistency to remain in AS [13]. Given the efficacy of mpMRI in detecting clinically significant PC, the hypothesis arises that certain patients with ISUP GG 1 prostate cancer on initial biopsy, but high prostate imaging and reporting data system (PI-RADS) scoring may harbour more aggressive cancers. For these patients, AS would result in a higher rate of discontinuation.

This study aims to identify parameters which predicted higher-grade PC in initial mpMRI and to correlate the MRI parameters with disease progression and upgrading of ISUP grade group at follow-up.

## Materials and methods

### Study design

The study was approved by the local ethics committee (study number 5910R). Each participant provided written consent before inclusion. The patient cohort included men who underwent mpMRI and were diagnosed with ISUP GG 1 through MRI/TRUS-fusion biopsies over a period from March 2014 to August 2021. These procedures were all carried out within our facility. Following the imaging, each patient received an MRI/TRUS-fusion biopsy, typically involving two targeted samples per identified lesion, complemented by a set of twelve systematic samples. Eligibility for the study required that patients be initially diagnosed with low-risk PC at our institution, with no prior treatment, and with comprehensive records of both MRI and biopsy procedures.

### Data abstraction

The classification as ISUP GG 1 referred to the histopathology from the initial targeted or systematic biopsy. Clinical and biopsy information comprised age, PSA levels, ISUP grade group, and the percentage of PC infiltration per core. MRI parameters included PSA density (PSAD), PI-RADS v2.1 score, localisation of the index lesion (IL) in either the peripheral zone (PZ) or transitional zone (TZ), largest diameter of IL, contact length to the prostatic pseudocapsule (LCC), MRI T-stage, ADC value, and focal dynamic contrast enhancement (DCE) positivity of the IL. All patients were primarily treated either with AS, radical prostatectomy (RP) (with/without pelvic lymph node dissection), or radiotherapy (RT) (with/without androgen deprivation therapy). For AS, patients followed the PROMM-AS protocol for AS: PSA value was measured every 3 months. If PSA remained stable, further mpMRI were performed after 12 and 24 months. If PSA raised ≥ 0.75 ng/mL, mpMRI was conducted earlier. If follow-up mpMRI showed stable disease (PRECISE score 1–3), the patient did not undergo re-biopsy but remained on PSA measurement. In cases with progression at mpMRI (PRECISE score 4–5), MRI/ultrasound fusion-guided biopsy was performed. If histological results revealed ISUP GG upgrade, the treatment strategy was evaluated [14]. Follow-up after RT or RP included periodical PSA testing every 3 months. Biochemical recurrence after RP was defined as an increase in serum PSA levels above 0.2 ng/mL [15].

### MRI imaging

The MR imaging was performed on 3 Tesla systems, utilising either a 60-channel phased-array surface coil or an 18-channel phased-array surface coil combined with a 32-channel spine coil. The imaging protocol adhered to the PI-RADS v2.1 standards, incorporating T2-weighted sequences in three orthogonal planes (axial measurements: 0.5 × 0.5 × 3.0 mm, field of view at 130 mm), diffusion sequences (using both z-EPI and rs-EPI methodologies with voxel dimensions of 1.4 × 1.4 × 3.0 mm and b-values ranging from 0 to 1800 s/mm^2^), along with dynamic contrast sequences (T1-weighted, with voxel variation from 0.8 mm to 1.5 mm cube, a total scanning duration of 3 min and interval resolution set at 7 s) [16]. ADC maps were produced via the system’s built-in monoexponential model processing. To guarantee the highest imaging standards, all examinations were rated according to PI-QUAL (version 1) [8].

### Biopsy procedure and histological examination

Under the guidance of a combined MRI/ultrasound system facilitating elastic fusion, urologists with at least 5 years of experience in fusion biopsies executed both targeted and systematic 12-core sampling using an automatic device. Suspicious regions identified through MRI underwent sampling with two additional cores. The subsequent tissue analysis adhered to the ISUP 2014 criteria, with radical prostatectomy samples providing conclusive histological reference in cases of biopsy discrepancy. RP specimens were evaluated for extraprostatic extension (pT3a/pT3b), margin status (R0/R1), and lymph node metastases (pN0/pN1). For repeated biopsy, we used either MRI/TRUS-fusion biopsy or MRI inbore biopsy in cases with unfavourable location of the suspicious lesion.

### Image interpretation and data analysis

MRI was evaluated prospectively in clinical routine supervised by an experienced radiologist (L.S. with 12 years of reading prostate MRI). Two experienced radiologists (M.B., with 5 years of experience in reading prostate MRI, and L.S.) reviewed the imaging datasets in a consensual approach, blinded to the pathological data. Prostate dimensions were ascertained using volumetric software (DynaCAD, Philips Healthcare), which also informed the calculation of PSAD by correlating serum PSA with prostate volume. The PI-RADS v2.1 framework was utilised to classify the imaging findings, with particular attention to lesion localisation, dimensions, and relevant anatomical features. MRI-based staging included assessments of extraprostatic involvement, especially focusing on extraprostatic extension (T3a) (cancer beyond the prostate pseudocapsule ≥ 3 mm) or seminal vesicle infiltration (T3b). ADC readings were measured via placement of a circular ROI within the index lesion, and DCE results were interpreted based on notable early or atypical contrast uptake within the suspect/correlating lesion. Additionally, MRI grading (mGG) was assigned ranging from 1 (indicative of low-grade PC) to 3 (suggestive of high-grade PC) as previously published [3]. The grading system is based on various MRI characteristics that predict the aggressiveness of PC. These include in particular DWI characteristics such as ADC value and high b-value signal intensity, T2/DCE appearance like infiltrative cross-zonal growth and T3 stage (Supplementary Table 1). Last, in case of negative targeted biopsy, we differentiated patients with targets in the same area as systematic biopsy revealed PC (in-field positive) from those where the described area was distant from the positive systematic core (out-of-field positive). For example, in the case of a positive systematic biopsy core in area 8 (PZpl base left) following a standard 12-core biopsy scheme, the core was evaluated as in-field positive if the lesion was described at MRI in the same region (PZpl base left).

### Statistical analysis

Statistics were performed using IBM SPSS® Statistics (Version 29, IBM Corp). *p*-values < 0.05 were defined as statistically significant. Wilcoxon signed-rank test was performed to compare continuous data; chi-square test was performed to compare categorical data. We conducted a univariate and multivariate logistic regression analysis to determine significant predictors of high-risk disease.

## Results

### Study population

A total of 156 patients with histologically proven ISUP GG 1 PC in MRI/TRUS fusion-guided systematic plus targeted biopsy at baseline were finally included. All patients had a PI-RADS v2.1 classification ≥ 3 (Table 1). Eighteen patients had a PSA ≤ 4.0 ng/mL before biopsy. Patients with PSA ≤ 4.0 ng/mL were referred to MRI either due to clinical suspicion of prostate cancer (e.g., digital rectal examination*, n* = 6 or positive family history*, n* = 2). A total of 117 patients showed PC suspicious areas in PZ and 29 in TZ. For ten patients, the suspicious area was located at the anterior stroma. MRI suggested extracapsular extension or seminal vesicle infiltration (cT3) in 16 of 156 patients. The mean percentage of infiltration in biopsy cores was higher for targeted biopsy than for systematic biopsy (mean 24% ± SD 28% vs. mean 17% ± SD 21%).

**Table 1:** Baseline parameters

**Patients** (*n*)		156
**Age** in years; median (IQR)		65 (58–73)
**PSA** in ng/mL; median (IQR)		7.4 (5.0–10.2)
**PSAD** in ng/mL/cm^3^; median (IQR)		0.25 (0.16–0.42)
**PI-RADS v2.1** (*n*)	3	26
	4	92
	5	38

*PSA* prostate-specific antigen, *PSAD* prostate-specific antigen density, *IQR* interquartile range

**Fig. 1:**
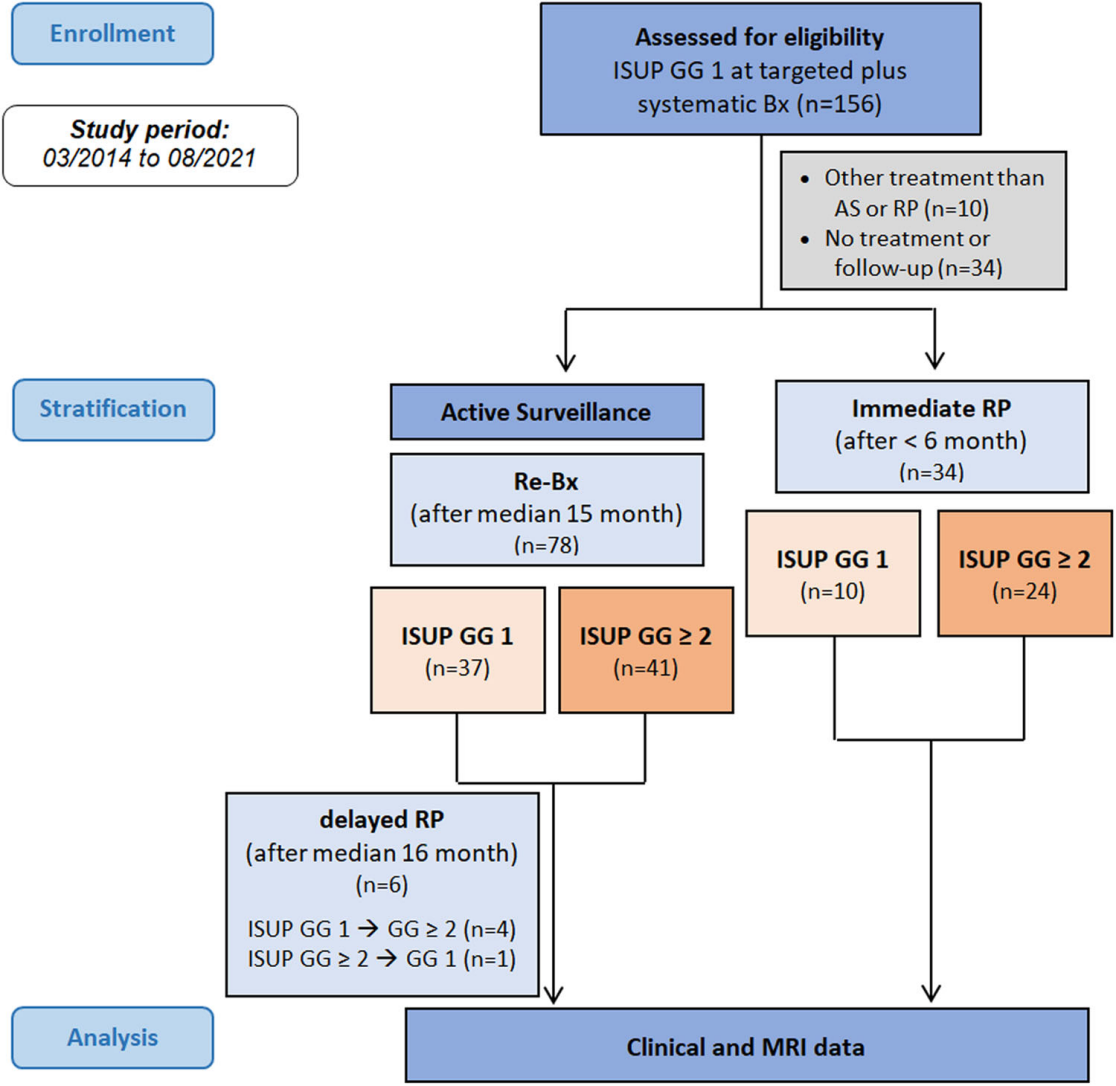
Flowchart of study design and patient selection

Follow-up data were available for 114 of 156 (73%). PC-related death was the case in one patient (0.6%). First-line treatment was AS in 100 patients, RP in 34 patients and RT in 10 patients. In twelve patients, no further information about tumour therapy was available. A CONSORT flowchart of the study population is shown in Fig. 1.

In 135 of the patients (87%), the initial positive biopsy cores originated from targeted biopsies in MRI-suspicious lesions. In 21 of 45 cases, the biopsy core with tumour detection in the systematic biopsy was distant from the suspicious area detected by MRI. CsPC was found in 3 of 21 cases, where the detected tumour was out-of-field (all ISUP 2). In the remaining 24 patients, the positive systematic core originated from the same area that was suspicious on MRI and was defined as in-field positive.

### Differences between confirmed vs. upgraded ISUP group

A total of 112 patients had a follow-up histopathological examination, either by a repeat biopsy (in AS setting to confirm low-risk PC) or RP specimen; 45% had confirmed ISUP GG 1 PC, 31% of the patients exhibited an upgrade of their ISUP GG at biopsy, and 27% exhibited csPC at histopathology of the RP specimen. Three quarters had an ISUP GG 2 PC, while one quarter harboured high-risk PC (ISUP GG 3–5 PC). Both groups did not differ significantly in age, PSA, or PSAD.

Low-risk tumours with confirmed ISUP GG 1 PC had a significantly lower percentage of infiltration in biopsy cores (combined targeted and systematic) at initial biopsy (*p* < 0.05). In addition, the PC was detected outside the biopsy field of the targeted core in 11 of 47 cases and was invisible at imaging, while in 36 cases, the area was visible on MRI and was addressed by targeted biopsy. For the group with ISUP GG upgrading, 3 of 65 lesions (5%) were invisible on MRI and only detected by systematic biopsy distant to the suspicious MRI lesion. For this parameter, both groups differed significantly (*p* = 0.03).

**Fig. 2:**
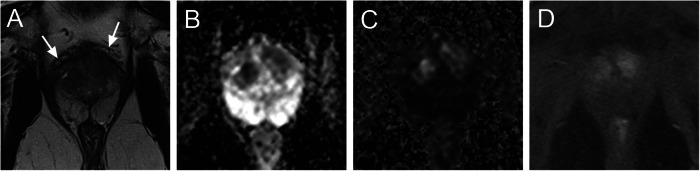
A 64-year-old patient with elevated PSA of 11 ng/mL; prostate volume was 53 mL, and PSAD was 0.21 ng/mL/mL. At bilateral apical transitional zone (**A**), there is a suspicious area with ADC value 707 × 10^−6^ mm^2^/s (z-EPI; ZOOMit) (**B**) and length to prostatic pseudocapsule of 11 mm. The high b-value image show a corresponding increased signal intensity (**C**) and DCE is positive (**D**). Lesion was categorised as PI-RADS 4 and mGG 3. Initial biopsy revealed ISUP 1 prostate cancer with infiltration of 80%. Re-biopsy revealed ISUP GG 2 prostate cancer in the right apical anterior lesion

**Fig. 3:**
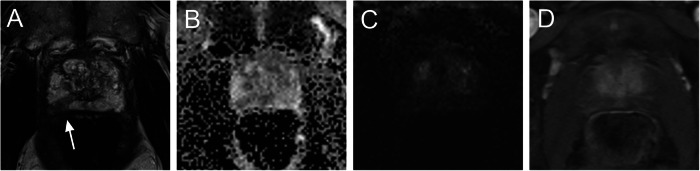
A 72-year-old patient with elevated PSA of 6.23 ng/mL; prostate volume was 56 mL and PSAD was 0.11 ng/mL/mL. At right apical peripheral zone (**A**), there is a focal area with ADC value 1060 × 10^−6^ mm^2^/s (rs-EPI; RESOLVE) (**B**). On the high b-value image there is no clear corresponding signal increase (**C**) and DCE is negative (**D**). Lesion was categorised as PI-RADS 3 and mGG 1. Initial biopsy revealed ISUP 1 prostate cancer with infiltration of 5%. Re-biopsy confirmed ISUP GG 1 prostate cancer

Regarding imaging parameters at univariate analysis, ISUP GG ≥ 2 cancer showed significantly higher mGG for upgrading at biopsy and RP (≤ 0.01), lower ADC values (*p* = 0.04) and exhibited enhancement on DCE more often (*p* = 0.02) (Table 2). Furthermore, LCC differed significantly between both groups (*p* = 0.04). Examples of cases with confirmed ISUP GG 1 and upgraded to ISUP GG ≥ 2 in the follow-up are shown in Figs. 2 and 3.

**Table 2:** Subgroups of patients with confirmed ISUP GG 1 vs. upgraded to ISUP GG ≥ 2 PC at repeated biopsy and/or RP specimen

			ISUP 1	ISUP ≥ 2	*p*-value
**Patients** (*n*)			47	65	
**Age** in years; median (IQR)			65 (59–72)	64 (58–71)	0.55
**PSA** in ng/mL; median (IQR)			7.3 (5.7–9.9)	7.6 (5.0–11.0)	0.51
**PSAD** in ng/mL/cm^3^; median (IQR)			0.17 (0.12–0.21)	0.20 (0.12–0.27)	**0.04**
**ISUP GG in Re-Bx or RP** (*n*)		1	47	-	n/a
		2	-	47	
		3	-	13	
		4	-	2	
		5	-	3	
**Max. infiltration in biopsy core** in %; median (IQR)			15 (5–40)	25 (10–50)	**0.03**
**PI-RADS v2.1** (*n*)		3	9	6	0.53
		4	29	42	
		5	9	17	
**T2**	**Localisation**	PZ	28	37	0.61
		TZ	19	28	
	**PC diameter** in mm; median (IQR)		12 (10–15)	12 (10–14)	0.78
	**Contact length to pseudocapsule** in mm; median (IQR)		9 (0–14)	12 (8–17)	**0.04**
	**cT3 stage** in % (*n*)		6 (3)	11 (7)	0.11
**DWI**	**ADC value** in × 10^−6^ mm^2^/s; median (IQR)		836 (732–1101)	768 (689–949)	**0.04**
**DCE**	**Focal enhancement on DCE** in %		55	72	**0.02**
**mGG** (*n*)		1	15	4	**< 0.01**
		2	13	15	
		3	19	46	

*PSA* prostate-specific antigen, *PSAD* prostate-specific antigen density, *DWI* diffusion-weighted imaging, *ADC* apparent diffusion coefficient, *DCE* dynamic contrast enhancement, *IQR* interquartile range, *mGG* MRI Grade Group, *ISUP GG* International Society of Urological Pathology Grade Group

*p*-values <= 0.05 are considered to be significant and are given in bold

### Subgroup analysis of patients after RP

Considering patients with immediate RP after diagnosis (median time to RP 2.3 months (IQR 1.7–4.0 months)), 29% had confirmed ISUP GG 1 cancer, while 71% showed an upgrading. Seven of 34 cancers were ISUP ≥ 3, and pT3 stage was the case in 10 of 34 patients. All the patients referred to surgery directly exhibited mGG2 or 3, and cT3 stage was described at MRI in 26% (Table 3).

**Table 3:** Patients with immediate RP (< 6 months after PC diagnosis)

		ISUP 1	ISUP ≥ 2
**Patients** (*n*)		10	24
**Age** in years; median (IQR)		64 (63–70)	62 (55–69)
**PSA** in ng/mL; median (IQR)		8.3 (7.3–11.2)	9.1 (6.9–11.6)
**PSAD** in ng/mL/cm^3^; median (IQR)		0.22 (0.18–0.33)	0.22 (0.17–0.29)
**Max. infiltration in initial biopsy cores** in %; median (IQR)		60 (26–80)	40 (10–70)
**ISUP GG at RP** (*n*)	1	10	-
	2	-	17
	3	-	5
	4	-	1
	5	-	1
**pT stage at RP** (*n*)	2a	4	0
	2c	6	15
	3a	0	8
	3b	0	1
**R1** in % (*n*)		10 (1)	25 (6)
**PI-RADS v2.1** (*n*)	3	1	0
	4	5	15
	5	4	9
**cT3 stage** in % (*n*)		20 (2)	29 (7)

*PSA* prostate-specific antigen, *PSAD* prostate-specific antigen density, *IQR* interquartile range, *ISUP GG* International Society of Urological Pathology Grade Group

### Multivariate analysis

For multivariate regression analysis to distinguish between confirmed ISUP 1 and upgrading at repeat biopsy. A significant parameter to differentiate between both groups was mGG (Table 4).

**Table 4:** Multivariate logistic regression analysis

Parameter	B	Std. error	z value	*p*-value
Intercept	0.9000	2.9570	0.304	0.76
PSA	0.0011	0.0517	0.020	0.98
Infiltration Bx core	0.0064	0.0088	0.727	0.47
PSAD	0.0024	0.0426	0.143	0.79
LCC	0.0495	0.0306	1.614	0.11
ADC	0.0006	0.0015	0.381	0.70
Diameter	−0.0748	0.0576	−1.299	0.19
DCE positivity	0.7973	0.4823	1.653	0.10
PI-RADS	−0.2824	0.4796	−0.589	0.56
**mGG**	0.8460	0.4504	1.878	**0.04**

*PSAD* prostate-specific antigen density, *ADC* apparent diffusion, *LCC* length to prostatic pseudocapsule, *mGG* MRI grade group, *Bx* biopsy

*p*-values <= 0.05 are considered to be significant and are given in bold

## Discussion

Our cohort incorporates imaging findings in patients with initially detected low-grade (ISUP GG 1) PC at first MRI/TRUS fusion-guided biopsy. These cancers differ significantly in their imaging appearance in prostate MRI, showing discrepancies in certain parameters such as ADC values or PI-RADS scores. As many of these PC (55%) showed an upgrade at repeated histopathology, one aim of this analysis was to investigate MRI characteristics in terms of predicting higher-grade PC, which could imply earlier follow-up biopsy.

ADC values, in particular, are an excellent parameter for estimating PC aggressiveness and correlate negatively with the Gleason score [17–19]. Especially latest DWI techniques not only enable excellent detection on imaging, but may also give insights into tumour biology [3, 20]. In our cohort, we could also observe the ADC values of diffusion-weighted imaging differed significantly between the upgraded ISUP group and the confirmed ISUP GG 1 group. ISUP GG 1 findings at biopsy with remarkable ADC reduction should be considered with caution, especially in the case of negative targeted biopsy. This is also emphasised by 24 cases of negative, targeted biopsies in which the positive, systematic core was taken from the conspicuous MRI area; 25% of these patients exhibited ISUP ≥ 2 at repeated biopsy. DCE positivity was observed more often in ISUP GG ≥ 2 cases for both, RP and repeat biopsy. The role of DCE is discussed controversy [21]. Results from a large multicentre study might give more information about the role of contrast agent in cancer detection [22]. Higher-grade PC did not exhibit higher diameter in our cohort; however, there were higher rates of cT3 stages at imaging and larger LCC. This is suggestive of a more invasive behaviour and might also give a hint of the presence of aggressive disease. Efforts are already underway to use MRI as an additional criterion for patients before a final treatment decision is made. These algorithms and nomograms use MRI markers to estimate the course of the disease [23–25].

Another important point that we would like to address is that the cohort contains exclusively tumours that are visible on MRI. There is evidence that MRI invisible lesions tend to be less harmful and might not need immediate treatment [26–28]. The PRECISE criteria are designed to evaluate disease progression in imaging for patients at AS [29]. In the initial form, these criteria do not distinguish between MRI visible and invisible PC. As these two tumour entities differ in their prognostic outcome, this was taken into account in the updated version [30–32]. It is noteworthy that 79% of the non-visible tumours which were only found by systematic biopsies showed no upgrade in the repeated histopathology, whereas upgrade of MR invisible lesions was the case in only three patients. On the other hand, PC, which were only found by targeted biopsy, had significantly higher infiltration in biopsy cores and tended to harbour intermediate or high-risk disease at confirmation biopsy. Still, these patients were diagnosed with ISUP 1 PC at the initial biopsy. One possible reason might be the unfavourable locations of these tumours, especially far anteriorly or apically, which are more difficult to reach with transrectal MRI/TRUS fusion-guided biopsy.

By definition, the group described is a low-risk cohort, based on the initial histology with evidence of ISUP GG 1 PC. In most cases, AS would be the treatment of choice if it is accepted by the patient. AS are currently aimed at low-risk and in studies at intermediate-risk patients with small PC. However, even between low and intermediate-grade PC, there are certain differences in outcome and prognosis with regard to tumour progression [2, 33]. As some of the patients prefer immediate surgical treatment, we analysed a subgroup of exclusive patients who received immediate RP. Here, too, there was a significant proportion of upgrade in the histopathology with 71% ISUP ≥ 2. Compared to the literature, there are rates of 20–30% for upgrading at RP [34]. However, as the lesions described in the present cohort are predominantly visible on MRI, this might suggest a higher proportion of csPC. For all patients with second histopathology, we observed an upgrade to ISUP GG 2 in 41% and to ISUP GG 3–5 in 14%. This is a relevant part of patients with higher-grade PC in an initial low-risk cohort, which probably faces an increased risk of metastatic tumour disease or tumour-related death [4].

What is the right strategy in cases with discrepancies between histopathology and MRI results? Nyk et al stated that PI-RADS scores might already be sufficient to indicate immediate operative treatment [35]. Still, this was based on systematic biopsies alone and MRI performed after biopsy. In our study, the PI-RADS category differed significantly between confirmed ISUP 1 patients and patients with upgrading at repeated histopathology, taking together RP and confirmation biopsy. These cases should be re-evaluated interdisciplinary between urologists and radiologists if the initial biopsy diagnosed ISUP 1 prostate cancer. Next, mGG was the only independent parameter to predict ISUP upgrade at multivariate analysis in these patients. As this parameter incorporates many MRI derived markers and can estimate PC aggressiveness, it might be useful to consider in the case of an ISUP 1 cancer diagnosis [3]. Especially in cases where MRI revealed mGG3 lesions, early re-stratification biopsy should be considered (e.g., within 6 months). In addition, lesions that exceed the prostatic pseudocapsule are generally difficult to reconcile with an ISUP GG 1 PC [3, 4, 36, 37]. If the MRI reveals a cT3c stage, a re-evaluation of the case is appropriate. The same should be applied in cases in which MRI diagnoses a focal lesion suggestive of prostate cancer with PI-RADS category 4 or 5 but there is a negative targeted biopsy. Other parameters that should lead to a critical reflection of the diagnosed ISUP 1 PC are ADC values below 800 × 10^−6^ mm^2^/s, LCC above 12 mm, and a high infiltration in the initial biopsy core ≥ 50%, which may already indicate a large/relevant tumour volume.

Some limitations of this study warrant discussion, including its retrospective nature and single-centre design. First, all initial biopsies were performed as MRI/ultrasound fusion-guided biopsies, but there is still the risk of sampling error. Incomplete prostate segmentation or inaccurate lesion registration are the primary causes of missing clinically significant prostate cancer (csPC) [38]. Therefore, enhancing accuracy may be achieved through thorough evaluation of MRI quality, precise assessment of the MRI-conditional risk (mCSR), and leveraging advanced biopsy expertise. Second, mpMRI images were rated by two radiologists in consensus, therefore interobserver variability could not be assessed and consensus readings might have influence on accuracy compared to clinical practice, as two radiologists may achieve better results than a single radiologist. However, this approach can display the proof of concept with less influence of different experience level. Additionally, the rating might be influenced by local standards and preferences. This could probably bias the diagnostic performance of mpMRI, too. Third, even if some of the chosen parameters differed between the confirmed and the upgraded group, a definite distinction is merely possible based on this information alone. Other clinical factors should be taken into account. Finally, we can only suggest incorporating MRI grading into clinical decision-making before choosing a treatment strategy due to the retrospective design of our study. Prospective data is needed to confirm the real value and inter-reader variability of mGG to distinguish between different ISUP GG.

MRI data reveals important information about PC aggressiveness and should be incorporated into clinical decision-making when ISUP GG 1 PC is diagnosed on initial prostate biopsy, also after experienced MRI/TRUS-fusion biopsy approaches. In cases with clear MRI characteristics for a csPC (e.g., mGG 3 and/or PI-RADS 5, or clear focal PI-RADS 4 lesions on MRI), early re-biopsy (e.g., within 6 months) might be considered.

## Supplementary information

Supplementary Material

## Abbreviations

ADC: Apparent diffusion coefficient
DCE: Dynamic contrast enhancement
DWI: Diffusion weighted imaging
IL: Index lesion
ISUP GG: International Society of Urological Pathology Grade Group
LCC: Length of capsule contact
mGG: MRI grade group
mpMRI: Multiparametric magnetic resonance imaging
PC: Prostate cancer
PI-RADS: Prostate Imaging and Reporting Archiving Data System
PSA: Prostate specific antigen
PSAD: Prostate specific antigen density
PZ: Peripheral zone
RP: Radical prostatectomy
RT: Radiotherapy
TZ: Transitional zone
