# Supplementary material for: MRI characteristics predict risk of pathological upgrade in patients with ISUP grade group 1 prostate cancer
Source: Eur Radiol. 2024 Sep 13;35(4):2170–9. doi: 10.1007/s00330-024-11062-2 (PMC11913941; doi:10.1007/s00330-024-11062-2)
Supplement: Supplementary file 1 — Supplementary Material [file 330_2024_11062_MOESM1_ESM.pdf]

Supplemental Table 1: Definition of MRI grading groups (mGG) to assess the PC aggressiveness.

		MRI grading group		
		**mGG1**	**mGG2**	**mGG3**
**DWI**	**Hypointense signal on ADC** $[\times 10^{-6}\, mm^2/s]$	> 900 (rs-EPI)<br>> 800 (ss-EPI)	< 1000 (rs-EPI)<br>< 900 (ss-EPI)	< 900 (rs-EPI)<br>< 800 (ss-EPI)
	**Hyperintense signal on high b-value images**	0 or 1	0 or 1	1
		**and**	**and/or**	**and**
**T2 / DCE**	**Focal suspicious signal decrease on T2**	Discreet	Discreet, overlayed or clear	Clear
	**Multifocal or Cross zonal growth**	0	0 or 1	0 or 1
		**and**	**and**	**or**
**EPE (≥ cT3a)**		0	0	1

0 = no; 1 = yes

PCA = prostate cancer; T2 = T2 weighted imaging; DWI = diffusion weighted imaging; DCE = dynamic contrast enhanced MRI; ADC = apparent diffusion coefficient; rs-EPI = readout-segmented multi-shot echoplanar imaging; ss-EPI = single shot echoplanar imaging; EPE = extraprostatic extension
